# Prolonged Classical NF-κB Activation Prevents Autophagy upon *E. coli* Stimulation In Vitro: A Potential Resolving Mechanism of Inflammation

**DOI:** 10.1155/2008/725854

**Published:** 2008-06-11

**Authors:** Silke Schlottmann, Franziska Buback, Bettina Stahl, Rainer Meierhenrich, Paul Walter, Michael Georgieff, Uwe Senftleben

**Affiliations:** ^1^Department of Anesthesiology and Intensive Care, University of Ulm, 89075 Ulm, Germany; ^2^Department of Oncology, Lombardi Comprehensive Cancer Center, Georgetown University, Washington, DC 20057, USA; ^3^Department of Dermatology and Allergic Diseases, University of Ulm, Maienweg 12, 89081 Ulm, Germany; ^4^Electron Microscopy Unit, University of Ulm, 89069 Ulm, Germany

## Abstract

Activation of NF-*κ*B is known to prevent apoptosis but may also act as proapoptotic factor in order to eliminate inflammatory cells. Here, we show that classical NF-*κ*B activation in RAW 264.7 and bone marrow-derived macrophages upon short E. coli coculture is necessary to promote cell death at late time points. At 48 hours subsequent to short-term, E. coli challenge increased survival of NF-*κ*B-suppressed macrophages was associated with pattern of autophagy whereas macrophages with normal NF-*κ*B signalling die. Cell death of normal macrophages was indicated by preceding downregulation of autophagy associated genes *atg5* and *beclin1*. Restimulation of macrophages with LPS at 48 hours after E. coli treatment results in augmented proinflammatory cytokine production in NF-*κ*B-suppressed macrophages compared to control cells. We thus demonstrate that classical NF-*κ*B activation inhibits autophagy and promotes delayed programmed cell death. This mechanism is likely to prevent the recovery of inflammatory cells and thus contributes to the resolution of inflammation.

## 1. INTRODUCTION

Cell death of inflammatory and immune cells is of particular importance for the homeostasis of the immune system and also during defense mechanisms against infectious micro-organisms. Modulation of phagocyte cell death by bacteria, however, is discussed as an important mechanism of pathogenesis. For example, certain bacteria such as *Shigella* and *Salmonella* are capable of escaping from intracellular killing after phagocytosis and eventually lead to phagocyte death [1]. On the other hand, low-virulent bacteria such as *Staphylococci*, *Streptococci*, or *Escherichia coli* (*E. coli*) are usually unable to survive in phagocytes and are cleared efficiently by neutrophils and macrophages. Notably, these pyogenic bacteria may also induce programmed cell death of macrophages subsequent to phagocytosis (phagocytosis-induced cell death, PICD) [2, 3]. Although the significance of this form of cell death is not clear, it is generally believed that an early elimination of activated immune effector cells such as macrophages may severely impair the clearance of infections, whereas the elimination of terminally differentiated phagocytes contributes to resolve the inflammatory process [4, 5].

During the last decades, cell death was attributed to either necrosis or apoptosis, regardless that this simplified dichotomic classification neglects the existence of untypical cell death forms [6]. There is evidence that several alternative death programs exist and that they crosstalk in multiple ways. Basically, two forms of programmed cell death (PCD) are discussed. Type I PCD, which is synonymous to classical apoptosis, is characterized by condensation of cytoplasm and chromatin, DNA fragmentation, and cell shrinkage into apoptotic bodies, followed by removal of the dying cells by phagocytes. In contrast, type II PCD, which is often referred to as autophagy, is less well characterized. At early stages of autophagy, the plasma membrane may change morphologically and blebbing can occur [7]. Typically, autophagic cells display an accumulation of double-membraned vesicles: the autophagic vacuoles (AVs) [8] which were initially described during amino-acid or serum starvation of cells. Therefore, autophagy can be interpreted as a survival supporting mechanism. On the other hand, AVs may also be used by different pathogens such as *Salmonella* or *Mycobacterium tuberculosis* to escape from phagosomes. This leads to replicative niches in the phagocytic cell itself and eventually induces killing of macrophages [1, 9]. It is not entirely clear whether autophagic activity of cells is the cause of death or is actually an attempt to prevent it [10]. Although AVs were described in the context of type II PCD, autophagy itself does not necessarily lead to cell death, but may serve as a protection mechanism against apoptosis [11]. In fact, cell death or survival seems to be the result of simultaneous but differentially pronounced antiapoptotic and autophagic signalling. So far, the molecular basis and signalling events accounting for the mutual relationship between apoptotic and autophagic mechanisms remain largely unexplored.

One of the most widely used cellular signal-transduction pathways in response to bacterial exposure is the NF-*κ*B (nuclear factor kappa B) pathway. Signalling via the NF-*κ*B pathway is crucial for the induction, maintenance, and subsequent downregulation of inflammatory responses and is also involved in the regulation of cell proliferation and survival [12]. Usually, classical NF-*κ*B activation via phosphorylation and subsequent degradation of its inhibitor I*κ*B*α* (inhibitor of NF-*κ*B *α*) results in inhibition of PCD due to increased transcription of antiapoptotic genes [13]. Moreover, complete absence of classical NF-*κ*B signalling regularly results in apoptosis [14, 15]. Currently, however, the dual function of NF-*κ*B with respect to the apoptotic process is becoming increasingly clear. There is experimental evidence that NF-*κ*B may act as pro- as well as antiapoptotic transcription factor depending on the cell type and the cellular environment [16]. Moreover, it was shown that the NF-*κ*B pathway may support proapoptotic mechanisms subsequent to its proinflammatory function [17].

So far, experimental evidence concerning negative regulation of NF-*κ*B signalling and its anti-inflammatory function in the context of bacterial infections is scarce. Recently, we have shown that phagocytosis of low-virulent *E. coli* by Raw 264.7 macrophages suppresses early phagocytosis-induced cell death via classical NF-*κ*B activation [18]. In the study presented here, we determined a new role of NF-*κ*B in cell death of Raw 264.7 macrophages and murine bone marrow-derived macrophages (BMDM) following short-term *E. coli* coculture. Using NF-*κ*B-suppressed and normal macrophages, we demonstrate that suppression of long-term activation of classical NF-*κ*B signalling upon bacterial challenge leads to extensive autophagic vacuolarization, improved survival and increased cytokine production upon LPS restimulation in NF-*κ*B-impaired macrophages. This work supports the hypothesis that signalling via classical NF-*κ*B inhibits autophagy by suppression of autophagy-related genes (*atg*) and thereby promoting cell death. We thus describe a new function of classical NF-*κ*B signalling in pathogen activated macrophages which inhibits “self-healing” autophagy thereby contributing to the resolution of inflammation.

## 2. MATERIAL AND METHODS

### 2.1. Cell culture and antibodies

Murine macrophages (RAW 264.7) were cultured in DMEM (Invitrogen) supplemented with 10% FCS (PAA), 1% Glutamax I (Invitrogen), and 0,02 mg/mL Refobacin (Merck) at 37°C in 5% CO_2_. Long-term culturing of transfected cells was performed with 200 *μ*g/mL geniticin (Invitrogen). Stimulation of cell cultures were carried out with 1 *μ*g/mL LPS (from Escherichia coli O26:B6, Sigma) or was performed by coculture of cells with 5 × 10 e6 E. coli (Strain Top10) for 1 hour, which correspond to a 1 : 100 diluted E. coli culture with an OD of about 0,6. Subsequently, E. coli was removed by washing 3 times with medium. To synchronize cells for cell cycle analysis, they were serum starved overnight. This serum starvation had no consequences of the occurrence of autophagy. Polyclonal antibodies against I*κ*B*α* (C21), p65 (C-20), c-IAP-2 (H-85), actin (C-11), beclin1 (H-30) were obtained from Santa Cruz. Additionally, antibodies for CD95L (transduction), p100/p52, mTOR, phospho-mTOR, p70S6K and caspase 3 (cell signalling), p50 and p-cJun (Abcam), p53 (R&D systems), and bax (upstate) were used. For bone marrow-derived macrophages (BMDMs), bone marrow from C57Bl/6 mice was flushed from femurs in DMEM (Invitrogen) supplemented with 10% FCS (PAA), 1% Glutamax I (Invitrogen), and 0,02 mg/mL Refobacin (Merck) and cultured in DMEM conditioned with L929 medium (30%). After 24 hours floating cells were recultured and BMDMs were used for experiments after 1 week. To inhibit prolonged NF-*κ*B activation in BMDMs, Pyrrolidinedithiocarbamate (PDTC, 10 *μ*M) was used 1 hour subsequent to the removal of E. coli. Medium exchange experiments were carried out by collecting 24 hours conditioned medium from E. coli-stimulated macrophages and adding it to nonstimulated Mock and SR macrophages. Cell death and morphology were examined during the following 36 hours period of incubation. For restimulation experiments, E. coli pretreated cells were collected after 48 hours, washed, and centrifuged. 1 × 10 e6 live cells (trypan blue exclusion) were stimulated with 10 U/mL IFN*γ* and 1 *μ*g/mL LPS (from Escherichia coli O26:B6, Sigma) for 24 hours and levels of IL-1*α* and IL-12 were measured in the supernatant by Elisa according to the manufacturers instructions (R&D systems, Minneapolis).

### 2.2. DNA constructs transfection and proliferation assay (MTT-assay)

The pcDNA3-I*κ*B*α*-super-repressor plasmid (SR) was kindly provided by Dr. R. Zwacka (University of Ulm, Germany). The cloned I*κ*B*α* sequence is mutated at Ser32/Ser36 to form a phosphorylation-resistant (A32/A36) I*κ*B*α* super-repressor (I*κ*B*α*-SR). Transfection, neomycin selection and subcloning as well as proliferation analysis of empty vector transfected (Mock)- and I*κ*B*α* (SR)-cells were described previously [18]. BMDMs were transiently transfected with an LC3-GFP fusion construct which was kindly provided by Professor T. Yoshimori (for details see [19]).

### 2.3. Protein extraction, immunoblot, electrophoretic mobility shift assay and RT-PCR

 For nuclear extract preparation cells, (2 × 10 e7) were washed two times in NP40-buffer (10 mM Tris-HCl, 10 mM NaCl, 3 mM MgCl_2_, 30 mM Sucrose, 0,5% NP40 pH 7.0), centrifuged (1500 g) for 7 minutes followed by two washing steps in CaCl_2_buffer (10 M Tris-HCl, 10 M NaCl, 3 mM MgCl_2_, 30 M sucrose, 0.1 mM CaCl_2_, pH 7,0). Nuclei were resuspended in lyses buffer (50 mM Tris-HCl pH 7.6, 250 mM NaCl, 3 mM EDTA, 3 mM EGTA, 1% TX100, 0,5% NP40, 10% glycerol) and lysed on ice for 30 minutes followed by centrifugation at 14.000 rpm for 30 minutes. Supernatants (nuclear extract) were collected and stored at −80°C. For total cell extracts, cells were directly lysed with lyses buffer. All steps were carried out at 4°C. Buffers were supplemented with 1 mM *β*-glycerolphosphat, 2 mM DTT, 1 mM PMSF, 10 *μ*M Leupeptin, 2 mM PNPP, and 0.1 mM orthovanadate. Immunoblots were performed as previously described [18]. For band shift assays, nuclear extracts (10 *μ*g) were incubated in a 10 *μ*L reaction for 30 minutes with 0.1 *μ*g/*μ*L polydIdC (Pharmacia) and 20,000 cpm ^32^P-*α*dATP labeled Hiv-*κ*B-site containing oligos in 1 mM DTT, 10 mM Hepes pH 7.6, 50 mM KCl, 6 mM MgCl_2_, 1.2 mM CaCl_2_, 1 mM DTT, 5% Glycerol. Complexes were separated in native 4% polyacrylamid gels for 3 hours, dried and exposed to AGFA Cronex 5 X-ray films. HIV-*κ*B-site containing oligonuleotides were described previously [18]. RNA was prepared according to Chomczynski and Sacchi [20]. 5 *μ*g RNA was reverse transcribed using superscript RNase H^−^(Invitrogen) according to the manufacturer's instruction. RT-PCR was performed using 0.125 *μ*g cDNA in a 35 cycles multiplex PCR reaction using one of the following mouse specific primer pairs and actin-specific primers in a multiplex approach under the appropriate cycling conditions: *atg5S*: acggagcggcctttcatc, *atg5R*: ggcttcggctgcattgc; *atg7S*: tggatacaagcttggctgctac, *atg7R*: agggtaagaccggtcaagtc; *beclin1S*: actggacacgagcttcaagatc, *beclin1R*: ctccaaacagcgtttgtagttc; *actinS*: ctacaatgagctgcgtgtgg, *actinR*: caggtccagacgcaggatgg.

### 2.4. Determination of dead and apoptotic cells

Cell death analysis was performed by means of DNA staining of permeabilized complete cells with propidium iodide (PI). Briefly, upon stimulation with E. coli as indicated 1 × 10^∧^6 cells were washed twice in PBS, fixed and permeabilized in ice cold 70% ethanol, washed in PBS and resuspended in 0.5 mL staining solution (0.2 mg propidium iodide (PI), 2 *μ*g RNAse in PBS). Flow cytometric analysis was performed within 1 hour. Expanded hypodense M1 population represents dead cells in general, while cells undergoing classical apoptosis normally accumulate in a distinct, narrow subG1 peak. More specifically than by PI intercalation of whole cells, apoptotic cells were determined by detection of PI intercalation in isolated nuclei according to Nicoletti et al. [21]. Apoptotic nuclei also accumulate as a subG1 peak. This method additionally permits cell cycle analyses. Furthermore, determination of dead cells, characterized by loss of membrane integrity, was performed by trypan blue exclusion. After staining with trypan blue, 300 cells were examined and dead cells were documented in percentage of cell death.

### 2.5. Visualization of autophagic vacuoles

Autophagic vacuoles can be stained by monodansylcadaverine (MDC) according to Biederbick et al. [22]. To detect AVs in BMDMs, cells were transiently transfected with an LC3-GFP fusion construct as described above. After stimulation as above, cells were scanned and photographed using confocal and light microscopy, respectively. Inhibition of autophagy could be assessed by treatment with 10 *μ*M 3-MA (3-methyl adenine) solubilized in 3% acetic acid. Electron microscopy was performed by high-pressure freezing and freeze substitution as described [23]. To quantitate AVs per cell, the total number of AVs per cell profile of 25 cells was determined for each condition.

### 2.6. Detection of differentially expressed genes

Differentially expressed genes in Mock and SR cells were detected by microarray analysis using murine topic-defined PIQOR™ immunology microarrays (Miltenyi Biotec) and the corresponding service. We carried out two biologically independent experiments using total RNA from E. coli stimulated macrophages. Stimulation was carried out for 12 and 36 hours as described above. RNA from unstimulated cells was used to set up the control experiment. SR cells were hybridized against Mock cells for each individual time point. Expression changes were expressed as ratio SR/Mock by dividing the corresponding fluorescence signal intensities. Thus value ±1 indicates no differential regulation, values >+1 and <–1, respectively, indicate corresponding up-and downregulation. No value means no expression of the particular gene or it has been removed from list due to less than 2-fold change in all spotted replicates and experiments.

### 2.7. Statistical analysis

The data were analyzed using student's *t*-test and Mann-Whitney-U test, respectively. A *p* < .05 value was regarded as statistically significant. All data were expressed as mean ± SE.

## 3. RESULTS

### 3.1. Expression of an I*κ*B*α* super-repressor in Raw 264.7 leads to reduced long-term NF-*κ*B DNA binding activity upon E. coli stimulation

**Figure 1:**
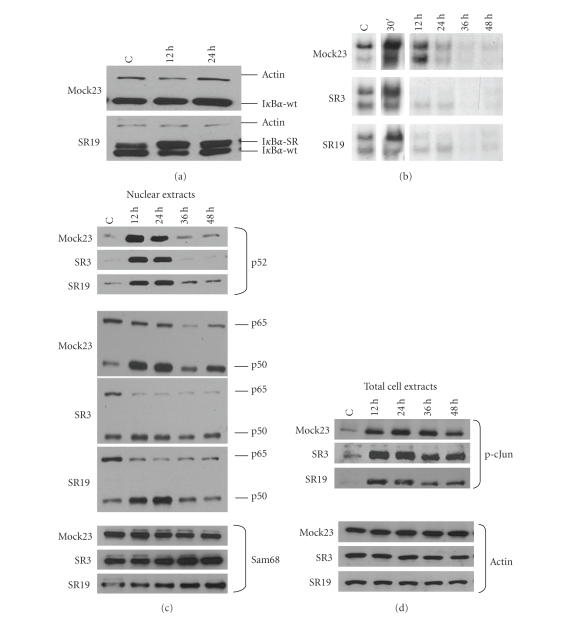
Impaired NF-*κ*B activity after E. coli stimulation. Mock and super-repressor (SR) transfected macrophages were cocultured with E. coli for 1 hour and subsequently cultured under standard cell culture conditions for the indicated time periods. C indicates untreated control cells. (a) I*κ*B*α* and I*κ*B*α*-SR are coexpressed in SR transfected macrophages. Immunoblot analyses were performed using total cell extracts. (b) Particularly, late NF-*κ*B DNA binding activity is impaired in SR-transfected macrophages. DNA binding activity was measured by EMSA analysis using 10 *μ*g nuclear extract incubated with ^32^P-labeled HIV NF-*κ*B binding site. (c) Immunoblot analysis shows accumulation of NF-*κ*B proteins p50, p65, and p52, respectively, in nuclear extracts (see Section 2). (d) JNK pathway is not affected by I*κ*B*α*-SR. Total cell extracts were tested for phospho-cJun expression by immunoblot analysis. Loading was controlled by detection of Sam68 in nuclear extracts and actin in total cell extracts. Data are representative of 3 independent experiments.

 In order to biochemically verify the inhibition of NF-*κ*B signalling by I*κ*B*α*-SR, expression of I*κ*B*α* (Figure 1(a)) and NF-*κ*B DNA binding activity (Figure 1(b)) in super-repressor (SR) macrophages were compared with empty vector transfected control cells (Mock). In SR cells, endogenous I*κ*B*α* and I*κ*B*α*-SR were coexpressed under baseline conditions (Figure 1(a)). Upon stimulation with E. coli, all screened SR cell clones initially revealed conserved, but moderately reduced NF-*κ*B activation within the first 30 minutes (Figure 1(b)). This is most likely due to the degradation of endogenous I*κ*B*α* (data not shown) leading to considerable NF-*κ*B release. At late time points lowed by a lack of NF-*κ*B activity in SR cells at late time points (Figure 1(b)). Thus in SR cells prolonged NF-*κ*B activation was abolished compared to Mock cells. To avoid clonal artefacts due to the insertion locus of the SR vector into genomic DNA, we compared several different clones with respect to NF-*κ*B activation. All SR clones displayed similar stimulation of DNA binding activity and similar kinetics of I*κ*B*α* and I*κ*B*α*-SR expression after induction with E. coli, respectively. Therefore, we selected two independent SR clones (no. 3 and 19) to serve as representatives for the suppressive SR function in all further experiments.

Expression of I*κ*B*α*-SR leads to reduced nuclear accumulation of p65 and p50 36 hours to 48 hours after stimulation compared to Mock cells (Figure 1(c)). NF-*κ*B suppression is limited to the classical pathway as no significant differences of nuclear p52 accumulation pattern could be detected in both Mock and SR cells (Figure 1(c)). Additionally, proliferation rates were not different in SR cells compared to Mock macrophages determined by means of an MTT assay (data not shown). Also, p-cJun levels were stable suggesting that other stress-induced signal transduction pathways were not affected by I*κ*B*α*-SR expression (Figure 1(d)). Thus due to merely partial blockade of initial NF-*κ*B activation this model serves to specifically investigate molecular and cellular effects that may primarily be affected by suppression of prolonged NF-*κ*B activity.

### 3.2. Suppression of prolonged NF-*κ*B activation is associated with increased cell survival

**Figure 2:**
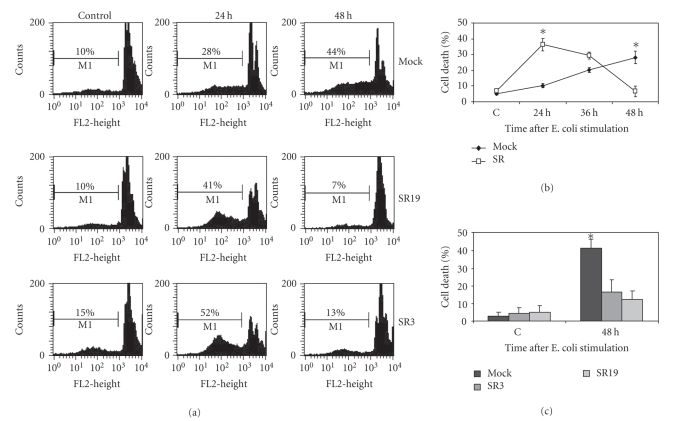
NF-*κ*B suppression leads to increased early cell death followed by improved recovery. Transfected macrophages were cocultured with E. coli as described above. After the indicated time periods, cell death was determined by means of flow cytometry (cell cycle analysis) (a), (b) and trypan blue exclusion (c). C indicates untreated control cells. (a) Percentages of dead cells were calculated from hypodense M1 population after propidium iodide staining of ethanol fixed cells. (b) Hypodense population of SR19 cells is significantly increased at 24 hours and significantly decreased at 48 hours following 1 hour E. coli stimulation. Data are shown as mean ± s.d. (*n* = 4).*p < .01. (c) Significant exclusion of trypan blue in SR cells at 48 hours upon E. coli ingestion. Trypan blue staining: percentage of dead cells was calculated as mean ± s.d. (*n* = 3). Each experiment results from counting 300 cells by means of light microscopy.*p < .01.

 Phagocytosis is a key mechanism of the innate immune system to combat invading pathogens. Phagocytosis of pathogens may lead to the induction of programmed cell death. We therefore examined the effect of E. coli phagocytosis on macrophage viability with respect to NF-*κ*B signalling. Cell death in response to short-term E. coli coculture was significantly different in normal compared to NF-*κ*B suppressed macrophages, as determined by FACS analysis. Early cell death rates of SR cells compared to Mock cells (24 hours) were significantly increased upon incubation with E. coli (Figures 2(a), 2(b)) suggesting a primarily antiapoptotic role of NF-*κ*B activation at an early stage upon bacterial challenge. It is conceivable that this mechanism reflects a basic property of NF-*κ*B activation to prevent death during cellular stress. Although the induction of cell death in SR macrophages was much more rapid than in Mock cells, the extent of death was balanced in both cell types after approximately 36 hours. Subsequently, remaining SR macrophages started to proliferate which is consistent with the recovery of this cell population, whereas Mock cells kept on dying. From 36 hours on, cell death rates of Mock cells clearly exceeded those of NF-*κ*B-suppressed SR macrophages. Similar observations could be made by trypan blue exclusion, exemplary shown for 48 hours (Figure 2(c)).

To rule out that cellular death of Mock and SR macrophages involves paracrine regulatory loops via secreted mediators, we tested conditioned medium of stimulated macrophages for its ability to cause cell death. We could not detect significant cell death rates in Mock macrophages incubated with Mock or SR-conditioned medium. Neither were we able to detect significant cell death in SR macrophages using Mock- or SR-conditioned medium (data not shown). In conclusion, NF-*κ*B-dependent regulation of cell death is not simply due to differences in mediators excretion (e.g., TNF*α*), but is rather associated with a cell-intrinsic death program.

### 3.3. Suppression of NF-*κ*B activation is associated with vacuole formation and subsequent recovery from E. coli-induced cell death

**Figure 3:**
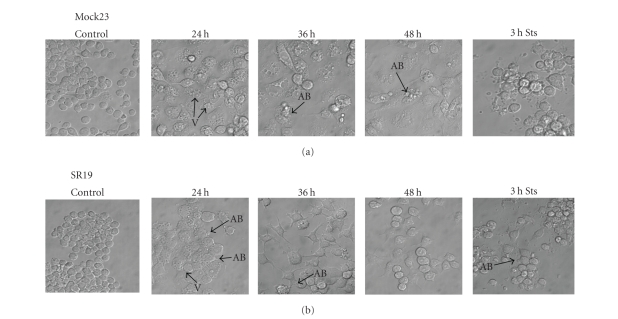
E. coli-induced cell death of macrophages is morphologically associated with extensive vacuolarisation and membrane blebbing. Cell death during 24 to 48 hours was microscopically examined in Mock and SR macrophages after 1 hour challenge with E. coli (described above). Extensive vacuolarisation (V) could be observed in Mock and SR cells at 24 hours. Apoptosis-like blebs (AB) were detectable at 24 hours in SR cells and at 36 hours to 48 hours in Mock macrophages. At 48 hours, physiological pattern dominated in SR cells. Mock cell numbers were clearly lower at 48 hours than SR cell number, therefore the image was taken from a cell-containing area to show differences in morphology. Staurosporine (Sts): cells were treated with 1 *μ*M Staurosporine for 3 hours to compare typical apoptotic morphological hallmarks. Images are representative of 2 independent experiments.

Regular NF-*κ*B activation is considered to be an essential mechanism to prevent programmed cell death during cellular stress [13]. In order to elucidate the mechanisms involved in early cell death of NF-*κ*B-suppressed SR macrophages and late cell death of normal Mock macrophages, we first examined the morphological structures of stimulated cells. Apparently, short-term E. coli exposure of Mock cells induced the formation of very large vacuoles within the first 24 hours (Figure 3 upper panel). Up to 36 hours, these vacuoles became larger and more numerous culminating in an enlargement of the whole cell and subsequent cell death after 36 to 48 hours. At 36 hours, cells also started to display membrane blebs as seen during apoptosis. SR cells displayed vacuole-like structures as well. In contrast to Mock cells vacuoles of SR, cells were smaller (Figure 3 lower panel). Membrane blebs in SR cells only occurred at 24 hours, but were absent at later time points. Stimulation-dependent vacuoles receded up to 36 hours and SR cells returned to a more physiological phenotype.

### 3.4. Cell death of macropages upon E. coli co culture is not due to apoptosis

The existence of membrane blebs suggests that an apoptosis-like mechanism could be responsible for the cell death of Mock cells. However, the morphological changes seen in Mock cells as well as in SR cells differ from the pattern seen during classical apoptosis. For example, we could not detect any pyknosis. In order to more specifically evaluate the form of cell death, we carried out a more sensitive PI staining of isolated macrophage nuclei according to Nicoletti et al. [21] and determined the expression of apoptosis regulating proteins.

**Figure 4:**
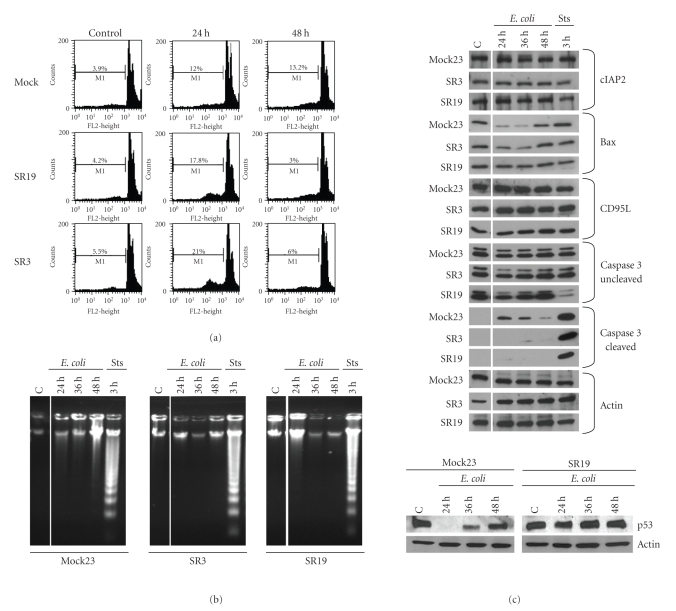
Involvement of apoptosis in PICD of macrophages. Transfected macrophages were cocultured with E. coli as described above. After the indicated time periods, apoptotic cell death was determined by measuring propidium iodide intercalation of isolated nuclei according to Nicoletti et al. [21] (a) as well as by preparing apoptotic DNA according to standard DNA preparation methods (b). Staurosporin-induced macrophage apoptosis (Sts, 1 *μ*M) was used for preparation of typical apoptotic DNA ladders. (c) Expression of apoptosis-associated proteins. Macrophages and bacteria were incubated as above and total protein extraction was prepared at indicated time points. Cell lysates were immunoblotted with antibodies as indicated. Actin was used as a loading control. Data are representative of 3 independent experiments.

It is well known that bacterial uptake can induce apoptosis of Raw macrophages [24]. However, the role of NF-*κ*B signalling in this context is not clear and may be pathogen-dependent [25]. As shown in Figure 4(a) temporary challenge with E. coli for 1 hour augments cell death within 24 hours in SR compared to Mock cells. A typical subG1-peak, however, could not be detected by staining of isolated nuclei [21]. This confirmed cell death rates previously determined with whole cell staining (Figure 2). At 48 hours, when rates of cell death of Mock cells clearly exceeded those of SR macrophages, we also failed to detect a circumscribed subG1-peak. Therefore, we investigated whether staining of nuclei correlates with nuclear fragmentation and expression of apoptosis-relevant proteins. Interestingly, neither typical apoptotic DNA fragmentation ladders nor significant up regulation of classic proapoptotic proteins could be detected at relevant time points in Mock or SR cells (Figures 4(b), 4(c)). Proapoptotic bax as well as p53 are downregulated in Mock cells 24–36 hours poststimulation but is induced again at 48 hours, whereas SR cells demonstrate stable levels as demonstrated by immunoblotting (Figure 4(c)). No difference for NF-*κ*B-regulated antiapoptotic cIAP2 (inhibitor of apoptosis 2) or proapoptotic CD95L expression could be detected. Yet, at 24–48 hours poststimulation effector caspase 3 is cleaved to a low extend in Mock cells but not in SR cells (Figure 4(c)). This may partially explain the occasional appearance of apoptotic morphology of Mock cells. As cleaving of the effector caspase 3 directs the cell to programmed cell death [26], our data suggest an indirect proapoptotic role for NF-*κ*B at late time points after bacterial challenge. This is in line with increasing bax levels at 48 hours in Mock cells. Robust levels of pro- and antiapoptotic proteins in SR cells upon bacterial challenge, however, suggest that blockade of classical NF-*κ*B signalling leads to stabilization of death pathways which prevents definite death of these macrophages.

### 3.5. Suppression of prolonged classical NF-*κ*B activity promotes autophagy of macrophages upon E. coli challenge

**Figure 5:**
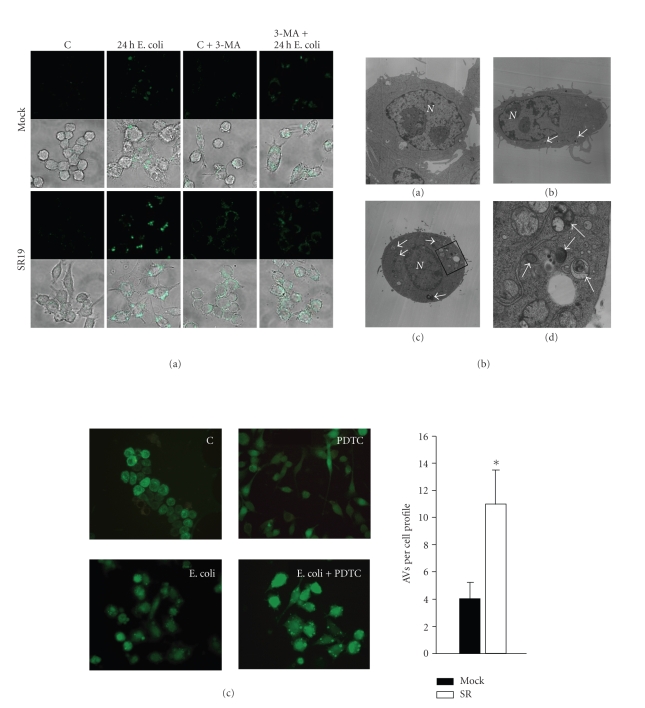
Suppression of classical NF-*κ*B causes autophagy upon E. coli phagocytosis. (a) Formation of autophagic vacuoles (AVs) in macrophages. Transfected macrophages were cocultured with E. coli in the absence and presence of 3-methyladenine (3-MA, 10 *μ*M), an inhibitor of autophagy, as described above. After 24 hours, the accumulation of the autophagy-specific dye monodansylcadaverine (MDC) in autophagic vacuoles was examined by confocal fluorescent microscopy. Note that unstimulated macrophage cultures may contain round and slightly spindle-shaped cells with a few small extensions. (b) *left*, (a)–(d), representative electron micrographs of control SR macrophages (unstimulated, a, 4000x), Mock macrophages at 24 hours poststimulation ((b), 4000x), and SR macrophages 24 hours subsequent to E. coli stimulation ((c) 4000x, (d) 20.000x). *N* = nucleus, the arrows denote AVs. *Right*, the total number of AVs per cell profile was determined 24 hours subsequent to E. coli stimulation. Results demonstrated are the mean ± s.d. of 25 profiles for Mock and SR cells.*p < .01. Data are representative of 3 independent experiments. (c) Autophagy is induced in BMDMs treated with the NF-*κ*B inhibitor PDTC. BMDMs were transiently transfected with an LC3-GFP fusion construct and either cocultured with E. coli or not (c). Only those cells that were treated with E. coli and PDTC (10 *μ*M, see Section 2) exhibited autophagy-specific granular accumulation of LC3 (arrowheads). Data are representative of 3 independent experiments.

To address the mechanism underlying this form of cell death, we evaluated the nature of the vacuoles of Mock and SR cells. Monodansylcadaverine (MDC) is known to accumulate specifically in AV, the so-called “autophagosomes”. A granular staining pattern indicates the formation of AVs [22]. Labelling of E. coli-stimulated Mock cells with MDC predominantly resulted in diffuse staining pattern and only few dyed vacuoles after 24 hours (Figure 5(a)). Over the observation period of 48 hours, no accumulation of MDC in AV could be detected in Mock cells. In contrast, E. coli coculture of SR cells clearly caused extensive MDC accumulation in AVs upon 12 to 24 hours (Figure 5(a)). Subsequently, AV staining decreased at 36 to 48 hours poststimulation (data not shown). Appearance of AV thus closely correlated with the occurrence of PI-hypochrome cells. Treatment of SR cells with 3-methyladenine (3-MA), a well-known inhibitor of autophagy, resulted in diffuse staining of macrophages and AVs disappeared (Figure 5(a)). Electron microscopy confirmed these observations. At 24 hours post E. coli-treatment typical AVs could primarily be detected in SR cells (Figure 5(b)). Importantly, E. coli-treated BMDMs exhibit similar pattern. BMDMs were transiently transfected with LC3, a homolog of Apg8p essential for autophagy in yeast and meanwhile often used to specifically detect AVs [19]. 24 hours subsequent to E. coli challenge, BMDMs, treated with PDTC to inhibit NF-*κ*B 12 hours before this observation point, clearly demonstrated granular morphology which specifically indicates AVs. These patterns were not detectable in control BMDMs not treated with PDTC (Figure 5(c)). Taken together, these data suggest that classical NF-*κ*B activation upon bacterial challenge prevents autophagy of macrophages.

### 3.6. Upon E. coli stimulation a utophagy-related genes atg5 and beclin1 are stably expressed in NF-*κ*B-deficient cells but are suppressed in control macrophages

**Figure 6:**
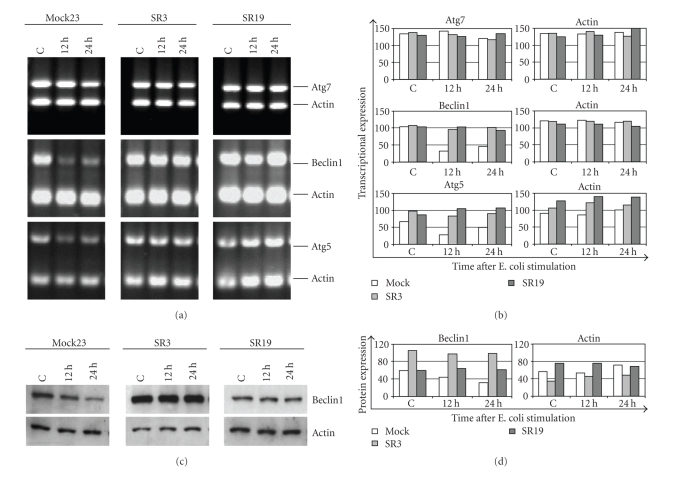
Classical NF-*κ*B activation by E. coli causes suppression of autophagy-related genes. Regular NF-*κ*B activity is necessary to suppress autophagy-related gene expression of atg5 and beclin1. RNA was prepared from control (c) and E. coli cocultured macrophages, reverse transcription was performed and cDNA was subjected to PCR for autophagy-related genes atg5, atg7, and beclin1. PCR was carried out as multiplex PCR with actin. Detailed information is described in Section 2. (a) RT-PCR of Beclin1, atg5, and atg7, respectively. (b) Densitometric analysis of gel image shown in (b). (c) Protein expression of beclin1 was determined by immunoblot analysis. (d) Densitometric analysis of immunoblot image shown in (d). All data are representative of 3 independent experiments.

It is known that there are at least three main *autophagy related genes* (*atg)* genes which are involved in the induction and formation of autophagic vacuoles in mammals: *beclin1*, *atg5,* and *atg7*. Products of these genes are essential for the process of autophagy, as their deficiency results in abolishment of the autophagic process [27, 28]. In order to investigate the molecular basis how NF-*κ*B inhibition promotes autophagy, RT-PCR analysis of the most relevant autophagy-related genes was performed. Determination of autophagy relevant genes showed a constant expression of *beclin1*, *atg5,* and *atg7* in SR cells during the formation of autophagic vacuoles (Figures 6(b), 6(c)). In contrast, Mock cells displayed a clear decrease of *atg5* and *beclin1* gene expression within 12 to 24 hours. Similarly, *beclin1* protein expression also decreased in Mock cells 24 hours after E. coli coculture, whereas it remains constant in SR macrophages (Figures 6(d), 6(e)). Therefore, prolonged classical NF-*κ*B activation is likely to suppress autophagy indirectly via inhibition of autophagy-relevant genes.

### 3.7. Differential expression of death and survival genes by microarray analysis

To elucidate the molecular mechanisms underlying the interrelation of the inflammatory capacity and cell death of macrophages, we performed gene expression analysis using *PIQOR immunology microarrays*. As shown in Table 1, the expression of several NF-*κ*B-responsive target genes was affected upon E. coli stimulation in SR cells. As expected, classical NF-*κ*B-deficient macrophages demonstrate suppression of proinflammatory genes. Also, *Ikba* and *nfkb2* were suppressed 2-fold in SR cells. Further, genes that were downregulated by classical NF-*κ*B deficiency encode for different death-related genes. Interestingly, antiapoptotic genes (A20, c-IAP1+2) as well as proapoptotic genes such like CD95, caspase 1, and cathepsin D are downregulated in SR cells suggesting a minor role of apoptosis in E. coli-treated and NF-*κ*B-suppressed macropahges. A second differentially regulated group of genes involves several transcription factors and tyrosine kinases, for example, src1. Most prominently suppressed are SOCS factors, ATF3, and IRAK-M. They are involved in cytokine signalling. In nearly all cases, differential regulation of genes became apparent only upon short-term challenge with E. coli. This explains the lack of differences in normal growth behavior between unstimulated Mock and SR cells (data not shown).

A third group of genes significantly upregulated upon NF-*κ*B inactivation and E. coli challenge consists of cell cycle- and proliferation-associated genes such as cyclin B2, cyclin D1, whereas PIM1 is downregulated. This indicates a modification of the proliferation properties of SR cells upon stimulation. Taken together, the data derived from microarray analysis support the idea that prolonged NF-*κ*B activity inhibits mechanisms that are involved in cellular function associated with survival and differentiation such as antiapoptosis, proliferation, and proinflammation.

### 3.8. Enhanced cytokine production upon LPS restimulation of E. coli-pretreated NF-*κ*B-deficient macrophages

**Figure 7:**
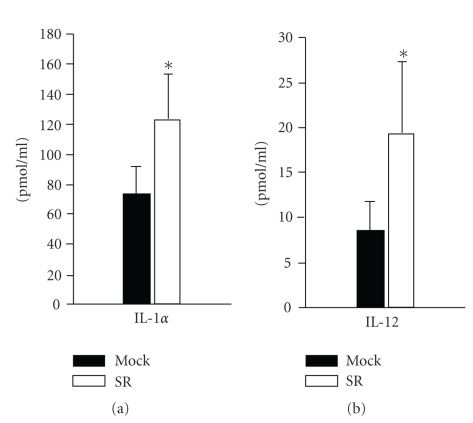
Improved recovery of cytokine production in NF-*κ*B-deficient SR macrophages. At 48 hours subsequent to E. coli stimulation Mock and SR macrophages (clone SR 19) were collected and live cells were restimulated with 10 U/mL IFN*γ* and 1 *μ*g/mL LPS as described in Section. 24 hours thereafter levels of IL-1*α* and IL-12 were measured in the cell culture supernatant by Elisa. Results shown are mean ± s.d. of 3 independent experiments.*p < .05.

To address the hypothesis that autophagy of NF-*κ*B-suppressed macrophages facilitates self-healing of these cells E. coli pretreated SR and Mock macrophages were restimulated with LPS/IFN*γ* at 36 hours and IL1*α* and IL12 production was measured. As demonstrated in Figure 7, production of these cytokines was significantly enhanced in SR cells. These data support the idea that autophagy leads to functional recovery of SR cells. Taken together, our results suggest that inhibition of classical NF-*κ*B activation eventually leads to the consolidation of proinflammatory macrophages. Thus classical NF-*κ*B inhibition at later time points is likely to prevent inflammatory resolution and may in turn promote chronic inflammation.

## 4. DISCUSSION

 In macrophages, we have shown that E. coli initially induces NF-*κ*B signalling to prevent cell death [18], whereas later it induces PICD, a mechanism that normally depends on caspase-3 activation [3]. This suggests a proapoptotic function of the classical NF-*κ*B pathway during later time points of macrophage activation. Indeed, suppression of prolonged NF-*κ*B activity in our SR macrophages is associated with reduced effector procaspase 3 cleavage and improved survival rates 48 hours subsequent to E. coli challenge compared to control macrophages. As expected, during earlier time points cell death rates were higher in NF-*κ*B-suppressed macrophages with caspase 3 being not induced. Most interestingly, this form of cell death was associated with autophagy. In this context, it is important to note that negative regulation of autophagic activity by caspases has been reported earlier [57]. On the other hand, it was shown that inhibition of autophagy triggers caspase-3 activation [58]. These data are in line with the observation that caspase 3 activation in Mock cells is associated with repression of autophagy. Several other facts point to the occurrence of autophagy in NF-*κ*B-suppressed macrophages that were treated with E. coli. Apart from typical AVs, the autophagic process may be accompanied by “apoptotic-like pattern”, such as membrane blebbing [7] as observed in our SR macrophages. Autophagy-specific MDC-positive AVs could not be detected in Mock macrophages throughout the observation period suggesting that activation of classical NF-*κ*B blocks the development of these AVs. Additionally, LC3, the yeast-homolog to atg6, which is proved to be specifically involved in the formation of autophagic vacuoles [19], also failed to accumulate in vacuoles of normal BMDMs subsequent to E. coli treatment. NF-*κ*B inactivation by PDTC, however, leads to specific accumulation in AVs which proves autophagy in these cells. It was also demonstrated that NF-*κ*B-suppressed Ewing sarcoma cells stimulated with TNF*α* exhibit autophagy [59]. This suggests that the repression of autophagy by classical NF-*κ*B activation constitutes a general cellular mechanism. Our observation of E. coli leading to downregulation of autophagy-related genes such as *beclin1* and *atg5* [28, 60] in NF-*κ*B-competent macrophages argues for a mechanism that acts via gene inactivation. This might occur through NF-*κ*B consisting of p50 homodimers, which were shown to repress gene transcription [61]. However, we speculate that NF-*κ*B-dependent inhibition of autophagy occurs indirectly. No relevant NF-*κ*B sites could be detected in the promoter regions of beclin1 and atg5 genes using computational analysis by means of MatInspector software [62]. In contrast, a putative binding site for p53, which is negatively regulated by NF-*κ*B, could be detected in the beclin1 promoter (position −1676). The involvement of p53 in the regulation of beclin1 expression is not known. However, the simultaneous suppression of beclin1, atg5, and p53 gene expression upon E. coli challenge in Mock macrophages suggests a negative direct or indirect regulation of autophagy relevant genes by NF-*κ*B. Presumably, the converse activity of NF-*κ*B and p53 [63] is important regarding the occurrence of autophagy in our system. In contrast to unaltered NF-*κ*B signalling in Mock cells, we could show by microarray and immunoblot analysis that NF-*κ*B-defective SR cells showed a lack of suppression in both p53 transcription and p53 protein expression after E. coli treatment. P53 in turn may prevent downregulation of autophagy relevant genes which leads to autophagy. Thus we conclude that classical NF-*κ*B activation upon bacterial ingestion normally inhibits autophagy at least in part via inactivation of autophagy genes. Taken together, our data demonstrate a new role of classical NF-*κ*B signalling in macrophages with the objective to suppress autophagy in the context of bacterial exposure.

 Hence late apoptosis is suppressed in NF-*κ*B-inhibited macrophages. How is it possible that suppression of apoptosis (PCD type I) promotes an autophagic process? It is known that predominating of antiapoptotic over proapoptotic molecules (e.g., as seen in bax/bak^−/−^ murine embryonic fibroblasts that cannot undergo classical apoptosis) favors the onset of autophagy upon etoposide treatment [64]. Conversely, it was also shown that inhibition of macroautophagy (PCD II) triggers apoptosis (PCD I) [58]. Accordingly, it is conceivable that autophagy and PCD are always induced in parallel. The outcome depends on prevailing factors. Autophagy and PCD may also be regulated by the same proteins. It was shown that Bcl-2 suppresses both apoptosis and autophagy [65]. One question that arises in this context is whether autophagic activity in dying cells supports cell death or is actually an attempt to prevent it. To our knowledge, no study has yet proven that autophagy induces cell death. In fact, there is considerable evidence that autophagic activity in dying cells might actually be an attempt to avoid death [66]. We believe that the accelerated recovery of macrophages devoid of prolonged classical NF-*κ*B activation has to be interpreted in this context. Thus the fate of the cells is likely to be determined by the balance of pro- and antiapoptotic signalling modulated by the extent of rescuing autophagy.

 It is not clear, however, what kind of cell death Mock macrophages execute 48 hours after E. coli challenge. Bacteria-induced death of cells may be linked to different forms of cell death, such as PCD I or necrosis [3, 67]. Despite activation of caspase 3 in these cells, the failure to detect signs of PCD type I, such as nuclear DNA fragmentation, is difficult to reconcile with classical apoptosis. It cannot be ruled out that phagocytosis of apoptotic cells by nonapoptotic macrophages masks typical apoptotic features. However, the uptake of trypan blue and the absence of a classical subG1 peak in an increasing hypodense propidium iodide positive population more likely reflects necrotic pattern. The fact that apoptosis and necrosis are often not clearly distinguishable is supported by the term “programmed necrosis” which recently emerged [68]. A considerable amount of evidence exists regarding the biological importance of programmed necrotic cell death, characterized by a disruption of membrane integrity [68]. During necrosis, cytosolic constituents spill into the extracellular space which may provoke an inflammatory response. This, however, may also promote an adaptive process to emerge a strong immune response [68]. This is in line with our data reported earlier, where we have shown that classical NF-*κ*B activation in macrophages prevents early PICD and eventually promotes T-cell activation [18]. At this point, however, it cannot be discriminated between the effects of partially suppressed early classical NF-*κ*B activation and its complete absence at late time points upon E. coli challenge. Thus it cannot be ruled out that all observations reported here and earlier [18] may derive from reduced initial classical NF-*κ*B activity. Moreover, inflammatory macrophage functions are directly regulated via the interactions of classical and alternative NF-*κ*B signalling [69]. To address this point, kinetic studies with specific IKK*β* and IKK*α* inhibitors need to be performed. Nevertheless, as classical NF-*κ*B activation per definition depends on I*κ*B*α* degradation, our studies give general insights into the role of the canonical pathway during inflammatory-associated cellular processes such as macrophage autophagy.

What is the biological importance of NF-*κ*B-mediated suppression of autophagy? PCD type I and subsequent ingestion of inflammatory cells by phagocytes is a physiological process for the removal of dying cells from sites of inflammation and essentially contributes to the resolution of inflammation in vivo [70]. Signals that promote leukocyte apoptosis are important for the resolution of inflammation and apoptotic cells themselves can support this process. It was shown that the phagocytic clearance of apoptotic cells by macrophages favors the release of TGF-*β*1 which in turn inhibits the proinflammatory activity of macrophages [71]. This process is thought to be NF-*κ*B associated [17, 72]. Here, we have shown that the absence of late proapoptotic NF-*κ*B signalling results in augmented survival of macrophages which is autophagy related. Furthermore, our data suggest that the downregulation of apoptosis- and upregulation of proliferation-regulating genes in NF-*κ*B-suppressed macrophages culminate in the recovery of inflammatory-differentiated macrophages. This is in line with increased cytokine production of NF-*κ*B-inhibited macrophages upon LPS restimulation. Thus we speculate that autophagy promotes the survival of inflammatory macrophage populations which may lead to the onset of prolonged or even chronic inflammation.

 In conclusion, our data outline an antiautophagic role of classical NF-*κ*B activation in macrophages that is likely to contribute to the resolution of inflammation. The death-supporting function of NF-*κ*B in this context has to be taken into account when anti-inflammatory therapies on the basis of NF-*κ*B-inhibition are considered. Further studies have to clarify whether inhibition of late NF-*κ*B activity in the course of infectious diseases protracts the recovery in vivo.

## Figures and Tables

**Table 1:** Selection of differentially expressed genes in Mock and SR cells. Two biologically independent experiments were carried out using *PIQOR* ™ *immunology microarray* (Miltenyi biotech). SR19 cells were hybridized against Mock cells. For each individual, time point gene expression changes were expressed as ratio SR19/Mock by dividing the corresponding fluorescence signal intensities expressed as mean value of four-spotted replicates. Data correspond directly to fold induction (+) and suppression (−) in SR cells, respectively.

Gene name	K	36h	Gene function	Unigene	Reference
*Apoptosis relevant genes*		
c-IAP1	−1,61	−2,86	Apoptosis (anti∼), caspases inhibition	Mm.14483	[29]
c-IAP2	−1,67	−3,13	Apoptosis (anti∼), caspases inhibition, intermediary in tumor necrosis factor alpha signalling	Mm.2026	[30]
A20		−8,33	TNF-*α* induced zinc finger protein A20, apoptosis (anti∼)	Mm.116683	[31]
CD95	−1,11	−10,00	Apoptosis (pro∼)	Mm.1626	[32]
Survivin	−1,03	1,54	Apoptosis (anti∼)	Mm.8552	[33]
Caspase 1	−1,54	−3,85	Apoptosis (∼pro), inflammation (pro∼)	Mm.1051	[34]
RANK	−3,57	−3,45	Differentiation of osteoclasts, promotes increased activity and survival of cells via antiapoptotic effect, induce production of proinflammatory cytokines	Mm.6251	[35]
Cathepsin D	−1,25	−2,94	Noncaspase protease, mediator of apoptosis, colocalizes with bid	Mm.231395	[36]

*Transcription and signal transduction *		
SYK	−1,10	−2,78	Signal transduction, tyrosine protein kinase (Spleen tyrosine kinase), G2M arrest, prevention of apoptosis	Mm.122843	[37]
SRC1	−1,67	−6,68	Signal transduction, proto-oncogene tyrosine protein kinase	Mm.22845	[37]
SRC2	−1,67	−2,57	Signal transduction, proto-oncogene tyrosine protein kinase	Mm.271665	[37]
ATF 3	+1,38	−11,11	Transcription factor	Mm.2706	[38]
c/EBP*β*	+1,25	−2,63	Transcription factor, CCAAT/enhancer binding protein beta	Mm.4863	[39]
NFAT2	+1,16	+1,66	Transcription factor control of T cell activation, differentiation, cell cycle, and apoptosis of T lymphocytes; transactivation of IL-4	Mm.329560	[40]
NF-*κ*B2	−1,09	−2,13	Transcription factor of the alternative NF-*κ*B pathway	Mm.102365	[41]
I*κ*B*α*	+1,31	−2,50	Signal transduction (Inhibition and retention of NF-*κ*B in the cytoplasm)	Mm.170515	[42]
Stat5A	+1,00	−1,85	Transcription factor induced by a variety of cytokines (e.g., IL-3, IL-5, GM-CSF), regulation of proliferation, differentiation and apoptosis of myeloid, erythroid and lymphoid cells	Mm.277403	[43]
SOCS		−5,88	Suppression of cytokine signaling	Mm.4592	[44]
SOCS-3		−11,11	Negative regulation of JAK/STAT pathways, suppression of cytokine signaling	Mm.3468	[44]
IRAK-M	−1,25	−7,69	Expressed in myeloid cells inhibits signaling downstream of IL-1R and Toll-like receptors (TLRs) via, cytokine-signaling (proinflammatory)	Mm.146194	[45]
TRAF-1	−1,47	−2,33	Signal transduction of TNF-receptor family, inflammation	Mm.239514	[46]

*Cell cycle and proliferation *		
P53	−1,05	+1,67	Tumor suppressor gene, cell cycle regulation, apoptosis regulation, DNA-strand break repair	Mm.222	[47]
Cyclin B2	+1,47	+2,45	Cell cycle regulation (G2/Mitotic specific) required for bipolar spindle formation in meiotic and mitotic cell divisions	Mm.22592	[48]
Cyclin D1	+1,15	+2,96	Cell cycle regulation (G1/S specific)	Mm.273049	[49]
PIM-1	−1,20	−2,56	Proto-oncogene serine/threonine-protein kinase, survival, proliferation, differentiation	Mm.328931	[50]

*Cytokines, chemokines, and receptors *		
IL-1*α*	−1,20	−25,00	Inflammation (pro∼)	Mm.15534	[51]
IL-6		−50,00	Proinflammatory, acute-phase reaction mediator, hybridoma growth factor and B cell stimulation) activates stat3, blocks apoptosis in cells during the inflammatory process	Mm.1019	[52]
TNF-*α* RII		−50,00	TNF-*α* signalling, survival of CD4 and CD8 T-cells during clonal expansion	Mm.235328	[53]
MIP-1*α* R		−12,50	Chemotaxis, induced by LPS	Mm.1282	[54]
CCL5	+1,11	−3,70	Chemotaxis, T-cell specific RANTES protein	Mm.284248	[55]
MCP-1	+1,53	−20,00	Chemotaxis	Mm.290320	[56]
